# Pulsed Radiofrequency for Postherpetic Trigeminal Neuralgia: A Case Report

**DOI:** 10.7759/cureus.28913

**Published:** 2022-09-07

**Authors:** Jaasiel Javier, Jon Wilton, Felice Galluccio, Abdallah El-Sayed Allam

**Affiliations:** 1 Department of Anaesthesia, Adventist Health Bakersfield, Bakersfield, USA; 2 Department of Anaesthesia, Dignity Health Mercy Medical Center Mt. Shasta, Mount Shasta, USA; 3 Rheumatology and Rehabilitation, Fisiotech Lab Studio, Firenze, ITA; 4 Pain Medicine, MoMaRC Morphological Madrid Research Center, Madrid, ESP; 5 Physical Medicine, Rheumatology and Rehabilitation, Tanta University Hospitals and Faculty of Medicine, Tanta University, Tanta, EGY

**Keywords:** chronic pain management, suicide disease, diagnosis and management of facial pain, tic douloureux, pulsed radiofrequency, postherpatic neuralgia, trigeminal neuralgia

## Abstract

Trigeminal postherpetic neuralgia has been reported to cause chronic headaches and facial pain. There are various modalities of treatment ranging from pharmacological to surgical treatment. However, these are frequently accompanied by undesirable side effects and minimal efficacy. Pulsed radiofrequency has been used to treat chronic pain but it is often seen as an unconventional treatment for postherpetic neuralgia. Nonetheless, the literature supports its use for alleviating pain with minimal complications. This case demonstrates that pulsed radiofrequency can successfully treat intractable pain secondary to postherpetic neuralgia affecting all three trigeminal dermatomes.

## Introduction

Postherpetic neuralgia is an intricacy of varicella herpes zoster in which the acute stage of neuritis causes nerve damage in the affected nerves, peripherally or centrally. The surrounding mucocutaneous tissues can also be affected by the inflammatory reaction triggered by the virus. Both reaction and tissue damage can cause hyperexcitability of the nerves to fire spontaneously [1].

A multimodal approach for the acute phase is usually implemented and this includes nonsteroidal anti-inflammatory agents (NSAIDs), opioids, nucleosides, and antivirals [1]. However, there is no definitive treatment thus far [1]. It is estimated that 10 to 15% of the acute phase leads to chronic conditions, also referred to as postherpetic neuralgia [1]. A single dose of live attenuated varicella-zoster virus (VZV) vaccine has been shown to reduce the incidence of postherpetic neuralgia [1].

Trigeminal neuralgia is an intense sudden pain accompanied by paroxysms in part or all branches of the trigeminal nerve and usually, the unilateral craniofacial dermatome is affected [2]. The trigeminal nerve encompasses most of the craniofacial sensory with some motor innervation and consists of three branches V1 (ophthalmic), V2 (maxillary), and V3 (mandibular) [2]. Pain is often described as a burning, shooting, and electric shock affecting various dermatomes that are innervated by trigeminal nerves [3]. A range of 4 to 13 out of 100,000 cases are reported yearly, and in 60% of cases, there is an involvement of only one branch, the maxillary or mandibular branch, whereas, in approximately 30% of the cases, both are involved [1,2]. The ophthalmic branch is rarely affected with only 4% of individuals reporting issues [3]. Trigeminal postherpetic neuralgia is more prevalent in females and the risk increases with age [1]. A multimodal pharmaceutical approach is also recommended to have a promising outcome, nonetheless, its effectiveness is only 25% [1-4]. This report will evaluate the efficacy of pulsed radiofrequency for the treatment of trigeminal postherpetic neuralgia.

## Case presentation

**Figure 1:**
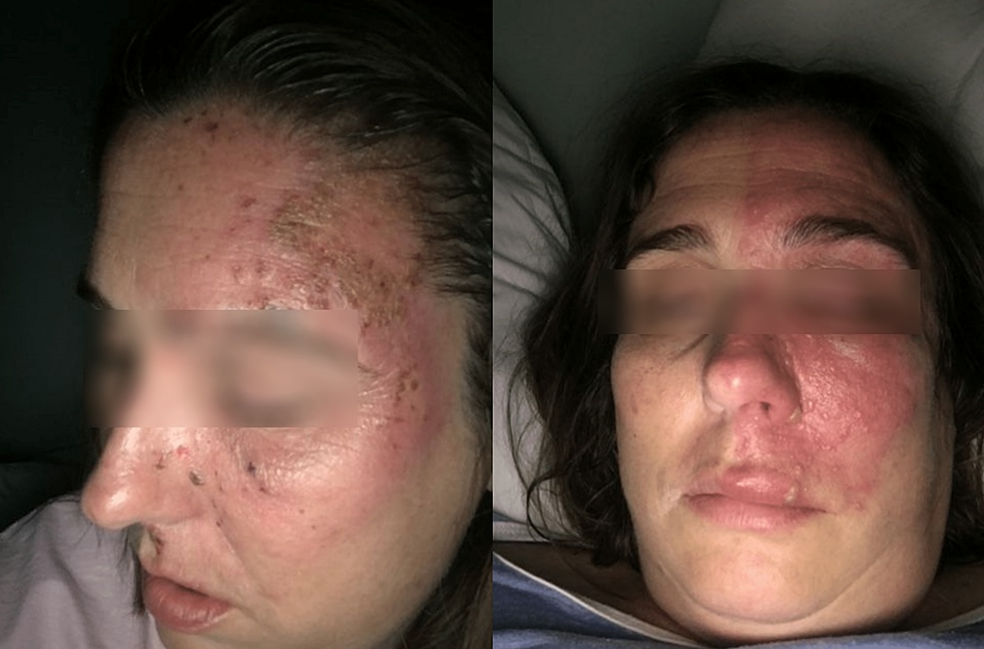
Herpes Zoster affecting the left V1 - V3 dermatomes

A 42-year-old, 82.1 kg, 177 cm female (BMI 26.21) with no known allergy presented with symptomatic trigeminal neuralgia secondary to herpes zoster. The patient reports a three-year history of unrelieved pain despite medications including carbamazepine and opioids prescribed at another treatment center. This also included a referral for stimulator placement which the patient declined. Upon presentation, she complained of incessant left-sided burning in the V1- V3 dermatomes numerically rated as 10/10 (numeric rating scale {NRS} 0-10). She also described allodynia and associated ocular pain (Figure 1).

The patient's medical history included intestinal intussusception, shingles, and postherpetic neuralgia. The patient had undergone many surgeries before (corneal graft, appendectomy, hysterectomy, bilateral knee surgery, right ulnar nerve transposition, tonsillectomy, right shoulder arthroscopy, exploratory laparotomy, cholecystectomy, and cesarean section) without anesthesia complications. Social history includes 1-2 drinks of alcohol per year, quitting tobacco use in 2004, and denies drug use. Her current home medications consisted of 400 mg acyclovir, 500 mg acetaminophen, 200 mg modafinil, 150 mg pregabalin, 50 mg tapentadol, and an estradiol patch of 0.1 mg.

Complete blood count, chemistry panel, and coagulation results were within normal limits. The preoperative vital signs were within limits and were as follows: heart rate 61 bpm, blood pressure 117/73 mmHg, temperature 36.5 degree Celsius, SpO2 100%, and respiratory rate of 10 breaths per minute. The patient complained of nausea preoperative but denied vomiting.

Upon arrival at the treatment area, anesthesia plans, risks, benefits as well as procedures were discussed and the informed consent was signed. Subsequently, noninvasive standard monitoring was applied, the left side marked and oxygen of 2 L/min via nasal cannula was provided. The patient was given 2 mg of midazolam and 20 mg of ketamine. The patient was sedated but capable of verbalizing extreme anxiety. An additional 20 mcg of dexmedetomidine was given and the patient was positioned with a right lateral decubitus with a bite block in position. Her face was then prepped with chlorhexidine and draped with sterile towels. A 15LA uSmart Linear Array Transducer, (3300 Terason, Burlington, MA, USA) was then covered with a sterile cover and the probe was then placed transversely over the gap between the zygomatic bone and mandibular condylar [5].

**Figure 2:**
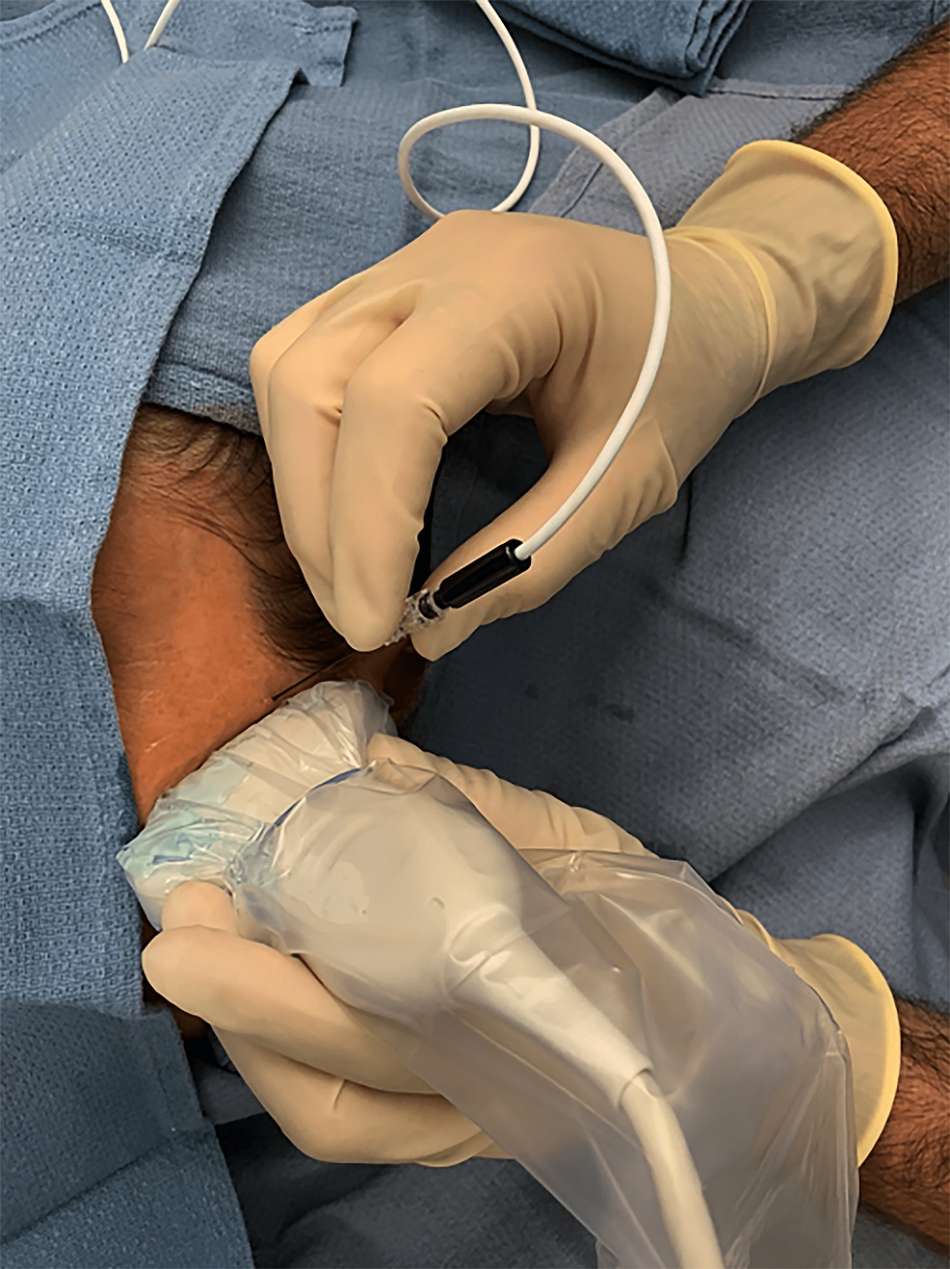
Ultrasuond guided PRF of Gasserian Ganglion PRF: Pulsed radiofrequency

Two milliliters of 1% lidocaine were used for skin localization and using a 100 mm Echobright needle (Avanos, Alpharetta, Georgia), the needle was inserted cephalad-direction via an out-of-plane technique (Figure 2). The needle was advanced until anterior to the lateral pterygoid muscle and inferior to the temporalis muscle [5]. The doppler was utilized with a needle to ensure separation from the artery. A mix of 2 mL of 1% lidocaine, 4 mL of 0.25% marcaine plain with 4 mg decadron was injected surrounding the trigeminal nerve. 

The patient’s pain was then reassessed and reported complete relief in the V1 and V3 dermatome but had residual infraorbital/V2 pain radiating to the inner canthus. Another 40 mg of ketamine was provided and the infraorbital area was prepped with chlorhexidine. The ultrasound probe was placed over the infraorbital notch and using a 22g needle, 1.5 mL of 0.25 % of bupivacaine with 1 mg of decadron was injected under ultrasound via in-plane technique. Post-procedure the patient reported complete resolution of pain which she had not experienced in three years. These injections were performed a second time 2.5 months later. Prior to the second injection, she reported her overall pain in the V1-V3 dermatomes was still reduced by 50%. After the second intervention, she once again had complete relief. These were considered successful test interventions as they both resulted in about two and a half months of significant pain relief.

Her pain returned 2.5 months after her second test injection and was rated as 5/10 (NRS 0-10). Pulsed radiofrequency (PRF) was initially discussed as a future treatment option. The three branches of the trigeminal ganglion were targeted for PRF. The left side was marked, noninvasive standard monitoring was placed and the patient was positioned right lateral decubitus with a bite block. The patient was given 2 mg of midazolam and 30 mcg of dexmedetomidine and the skin was localized with 2 mL of 1% lidocaine. The ultrasound probe was placed transversely over the gap between the zygomatic bone and mandibular condylar with the mouth open. Using the out-of-plane technique, an Avanos 100mm RFA introducer needle (Avanos, Alpharetta, Georgia) with a 4 mm active tip was inserted cephalad. An Avanos Nitinol 100mm RFA probe (Avanos, Alpharetta, Georgia) was then advanced until the anterior maxillary branch was stimulated using sensory stimulation down to 0.3 mA (Figure 2). This was accomplished using an Avanos Pain Management Generator. Two cycles of Pulse RF at 42 degrees Celsius with a duration of 180 seconds were performed. The needle was then redirected posteriorly while isolating the mandibular branch with sensory stimulation down to 0.3 mA. Two cycles of Pulse RF at 42 degrees Celsius for 180 seconds were provided. A doppler was utilized with a needle to ensure artery avoidance. A mixture of 2 mL of 1% lidocaine, and 4 mL of 0.25% marcaine plain with 4 mg decadron was then injected surrounding the maxillary and mandibular nerve.

**Figure 3:**
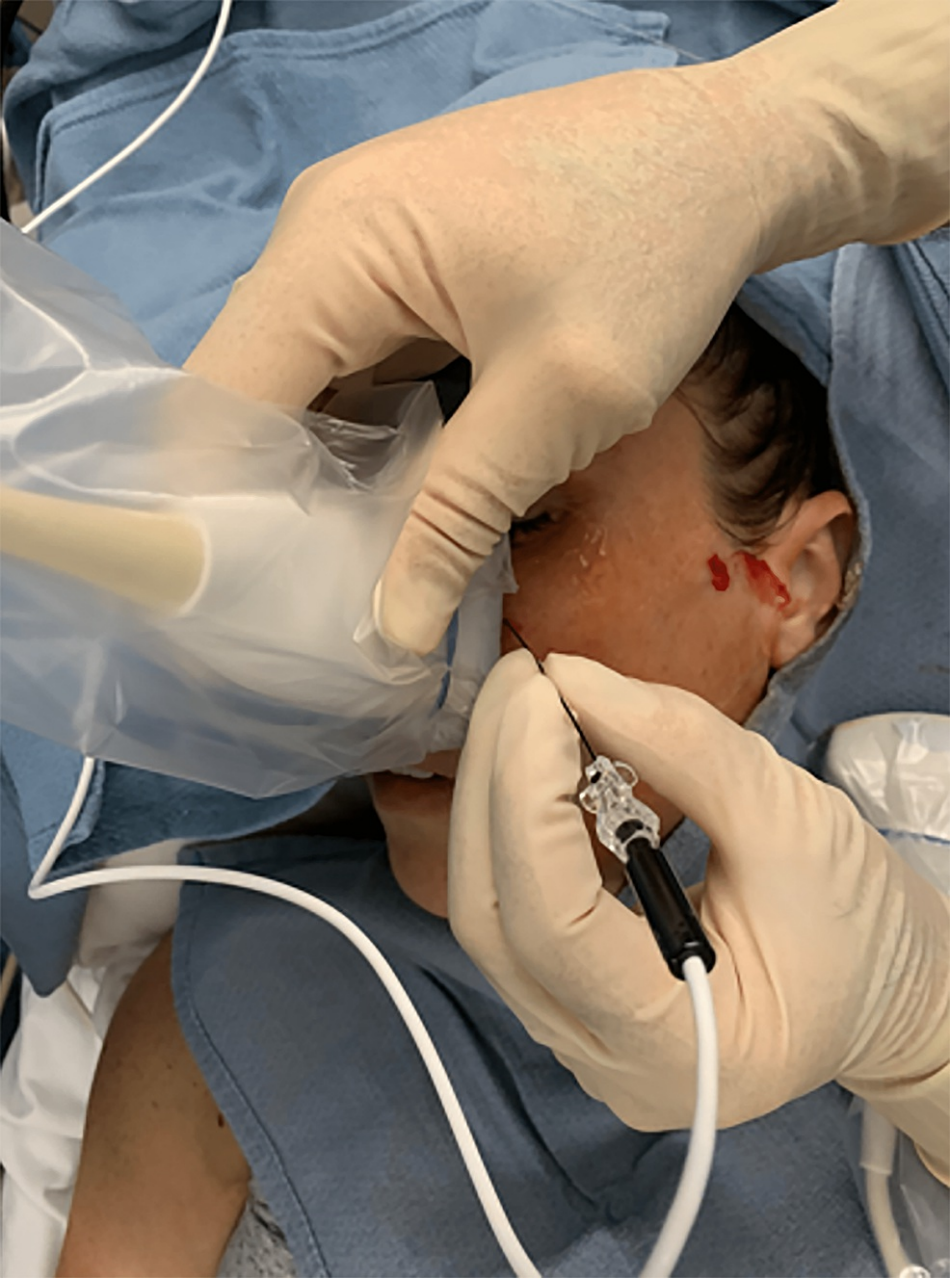
Ultrasound guided PRF of the infraorbital nerve. PRF: Pulsed radiofrequency

Once again, the patient complained of residual infraorbital/V2 pain. Another 2 mg of midazolam and 20 mcg of dexmedetomidine were given for anxiolysis. The ultrasound probe was then placed over her infraorbital notch where it was identified [6]. The skin was localized with 1 mL of 1% lidocaine using a 25 g needle under ultrasound. The same 100 mm RFA needle was then inserted, using an in-plane technique, until adjacent and parallel to the notch and 2 cycles at 42 degrees for 90 seconds were performed (Figure 3). One mL of 0.25% bupivacaine was then injected. The patient reported complete resolution of pain and an ice pack was provided.

Because of the coronavirus disease 2019 (COVID-19) outbreak, the patient who is a registered nurse (RN) was forced to wear an N95 face mask on a daily basis. Three months after initial treatment, the patient reported irritation secondary to the required mask. This pain was rated as 5/10 (NRS 0-10), and described as burning and sharp. Pulsed RF was repeated in the infraorbital branch for 3 cycles at 42 degrees Celsius for 120 seconds. At this time, the patient reported continued complete pain relief in both mandibular and ocular branches of the trigeminal nerve.

Ten months after the initial PRF, the patient reported having pain again in all the branches of the trigeminal nerve. This pain is again described as sharp and burning with allodynia and was rated as rating 4/10. Pulsed radiofrequency (PRF) of the Gasserian ganglion and infraorbital branch were again performed for two cycles of 180 seconds at 42 degrees Celsius. Four months later the patient still reports complete pain relief.

## Discussion

The anatomical approach to treating the trigeminal nerve can either be via the central Gasserian ganglion or via a more peripheral method. The peripheral branches can easily be identified and treated via the respective supraorbital, infraorbital, or mental foramina [5,6]. The technique that was used specifically in the study was detailed in the case report.

There are two categories of the trigeminal nerve, symptomatic and classical. Classical is idiopathic whereas symptomatic is secondary to a disease [2]. In this case study, the cause of trigeminal neuralgia (TN) is due to herpes-zoster and postherpetic neuralgia. There are two primary etiology theories [2]. One is nerve damage due to compression known as the neurovascular compression theory. This can result in damage atrophy, hypertrophy, or demyelination of the nerve causing the nerve to misfire causing pain [1,2]. Therefore, surgical interventions aim to alleviate the compression such as: percutaneous glycerol rhizotomy, percutaneous balloon compression, percutaneous stereotactic radiofrequency thermal rhizotomy, gamma-knife radiosurgery, partial sensory rhizotomy, and microvascular decompression [1,2]. However, these treatments have undesiring complications and a variable or poor efficacy in pain relief [4].

A second theory is known as the ignition hypothesis, claiming that structural changes of the nerve are the etiology of the pain [4]. This occurs as a result of the impulse of the axolemma being impeded due to damage to the nerve. As a result, a continuous action potential is fired making the nerve hyperexcitable. The electrical activity builds up and amplifies interaction in the neighboring structures resulting in pain. In this theory, the treatment aims to block the voltage-gated sodium channel to prevent the action potential from occurring via the utilization of pharmacologic treatments [4]. 

Anticonvulsants such as carbamazepine, and oxcarbazepine are first-line treatments for TN [3]. These treatments are usually combined with other pharmacotherapy modalities such as pregabalin, baclofen, and gabapentin. However, the efficacy of this approach is only 25% supplemented by pain control [4].

Aside from the aforementioned modalities for treating TN, pulsed radiofrequency (PFR) has been used for chronic pain treatment [7]. PFR uses high radiofrequency in a rotating current causing thermocoagulation without compromising nerve tissue. Nerve tissue damage occurs “between 60 and 80 degrees Celsius”, PFR thermocoagulation does not exceed 42 degrees Celsius and thus spares potential nerve damage [7]. Although its mechanism of action remains anecdotal, studies have shown it provides pain relief with minimal side effects [7].

In a retrospective study by Abd-Elsayed et al. [4], an analysis of eight different cases who have TN and were resistant to conventional modalities was performed. Participants were evaluated using a pain scale from 1-10 and evaluated for percentage of improvement through follow-up and duration of improvement tracked. The PRF was performed for 180 seconds from 40 to 60 degrees Celsius. All participants reported a significant decrease in pain of at least 50%, without any complications, p-value of 0.008. PRF facilitates the alleviation of pain for a duration of 91 days at least. The authors reported that three patients out of the 11 have full pain resolution postoperatively with just one session of PRF.

A similar finding was found by Chua et al. in a retrospective study of 36 participants with trigeminal neuralgia in which one participant died subsequently after surgical intervention [8]. Pain improvements were measured using a Likert scale and followed up at 2, 6, and 12 months intervals. All participants reported >80% of pain relief at all the follow-ups, and 55.9 % of the participants claimed to have >80% pain relief postoperatively without any reported complications. Out of the 34 participants, only 5 (14.7%) participants necessitated more than one treatment.

This is further echoed in another retrospective analysis by Luo et al. [9]. The study consisted of 28 participants with trigeminal neuralgia. Eleven of the 28 participants received PRF, while the remaining did not. All of the participants that went under PRF reported >50 % reduction in pain up to six months, however, six out of 11 participants needed more sessions after six months. The remaining participants who did not receive PRF reported poor outcomes necessitating PRF. It was reported that 17 participants did not relapse one year postoperatively.

Finally, the use of ultrasound, both for nerve block and PRF procedures, allows us to visualize all the anatomical structures correctly, facilitating not only the correct positioning of the needle [10] but also avoiding the numerous vascular structures and superficial and deep neurovascular that could cause unexpected complications [11].

## Conclusions

There is a consensus in the literature that PRF facilitates pain relief in trigeminal neuralgia without serious complications. PRF can cause edema, neural trauma, hematoma, and infection, however, none of these have been reported and these are minor in comparison to other side effects of the aforementioned treatment modalities. Although some require additional treatment, PRF can alleviate pain for about three months or so. Perhaps stronger levels of evidence are needed to solidify the efficacy of PRF further. These could come in the form of randomized controlled trials or larger cohort studies. Larger cohort studies may decrease confounding variables. Regardless, in this case report, pulsed radiofrequency was an effective modality. Since the last treatment, the patient remained pain-free and was able to stop pharmacological treatment.
